# SENSITIVITY AND SPECIFICITY OF THE MCDR FOR MCI DIAGNOSIS AMONG HISPANIC AND WHITE NON-HISPANIC OLDER ADULTS

**DOI:** 10.1093/geroni/igad104.1951

**Published:** 2023-12-21

**Authors:** Patricia Garcia, Lisandra Mendoza, Andres Duarte, Dilianna Padron, Michael Marsiske, Jacob Fiala, Ranjan Duara, Miriam Rodriguez

**Affiliations:** Indiana University School of Medicine, Indianapolis, Indiana, United States; Bay Pines VA Health System, Bay Pines, Florida, United States; Albizu University, Doral, Florida, United States; Central Virginia VA Healthcare System, Richmond, Virginia, United States; University of Florida, Gainesville, Florida, United States; University of Florida, Gainesville, Florida, United States; Mount Sinai Medical Center, Miami Beach, Miami Beach, Florida, United States; Indiana University Bloomington, Bloomington, Indiana, United States

## Abstract

**Objective:**

Greater functional impairment has been associated with higher rates of progression to dementia. Identifying sensitive functional measures that can detect early ADRD have immediate implications for prognosis, especially among ethnically diverse groups who are disproportionately affected. The modified Clinical Dementia Rating scale (mCDR) is a multiple-choice format assessment that assesses the five domains of the Clinical Dementia Rating scale. The current study aimed to compare sensitivity and specificity of the mCDR between White non-Hispanic (WNH) and Hispanic participants.

**Methods:**

The mCDR was administered in the patient’s primary language (English or Spanish) to 101 WNH Cognitively Normal (CN, n=20) and with MCI (n=81) and 159 Hispanic CN (n=32) and MCI (n=127) participants. Sensitivity to MCI diagnosis and Specificity to CN, using the mCDR “mean of means” total score, was quantified via Area under the Curve (AUC) calculated from a Receiver Operating Characteristic analysis.

**Results:**

Among WNH participants, the mCDR score demonstrated 78% Sensitivity and 90% Specificity (AUC = .88; CI95%= .81, .94). Among Hispanics, the mCDR showed 61% Sensitivity and 78% specificity (AUC = .73; CI95%= .64, .83). AUC was significantly worse for Hispanic participants.

**Conclusion:**

The mCDR is a tool that can be used by a trained psychometrist to facilitate focused answers and reduce administration time when assessing clinical functioning among dementia populations. It demonstrated excellent discrimination between CN and MCI among WNHs and acceptable discrimination among Hispanic individuals. Future research should further examine the utility of this tool for Hispanic older adults in early diagnosis of ADRD.

